# Bilateral superficial peroneal nerve entrapment secondary to anorexia nervosa: a case report

**DOI:** 10.1186/1749-7221-3-12

**Published:** 2008-04-27

**Authors:** Teoman Toni Sevinç, Aydıner Kalacı, Yunus Doğramacı, Ahmet Nedim Yanat

**Affiliations:** 1 Dept. of Orthopaedics and Traumatology, Mustafa Kemal University, Faculty of Medicine, Antakya, Hatay, Turkey

## Abstract

We report a case of severe weight loss secondary to anorexia nervosa causing bilateral superficial peroneal nerve entrapment in a young female patient who was treated successfully by bilateral surgical decompression.

## Background

Among entrapment neuropathies, superficial peroneal nerve (SPN) entrapment is relatively rare [1-8] and only a few bilateral cases have been reported in the literature [9,10].

Severe weight loss, as a result of anorexia nervosa, associated with common peroneal nerve entrapment is very rare [11-17] and SPN involvement alone has not been described in the literature published in English. Bilateral presentation is always related to systemic cause rather than local mechanical compression.

Herein we report a case of severe weight loss secondary to anorexia nervosa causing bilateral SPN entrapment in a young female patient who was treated successfully by bilateral surgical decompression.

## Case presentation

A 20-year-old, female university student presented to our outpatient orthopaedic clinic with a two month history of vague pain on the outer border of both legs, and numbness over the dorsum of the feet and big toes. Her symptoms were exacerbated by walking and running and partially relieved by elevation. She had to stop to rest after 30 minutes of walking because of intolerable pain.

There was neither history of trauma or surgery to the lower limb nor history of lower back problems. There was, however, a history of severe weight loss of (30 kg) during the previous six months and the patient was diagnosed with anorexia nervosa using criteria from the American Psychiatric Association's Diagnostic and Statistical Manual of Mental Disorders (DSM-IV-TR) and the World Health Organization's International Statistical Classification of Diseases and Related Health Problems (ICD).

Physical examination revealed bilateral tender points approximately 11 cm proximal to the ankle joint on the outer surface of the leg, Tinel sign was also positive bilaterally. There were sensory deficits on the dorsum of both big toes but no muscle weakness or abnormal reflexes.

Examination of the lumbar spine and lower limbs revealed no clinical abnormalities in the joints and there was neither suspicion of nerve root compression at the level of the lumbar spine nor nerve entrapment at the neck of the fibula.

Radiographic examination of the lumbar spine, legs and feet were normal and EMG studies were positive for bilateral entrapment neuropathy of the SPN proximal to the ankle joint with no abnormality of the common peroneal nerves or of the proximal nerve roots.

**Figure 1:**
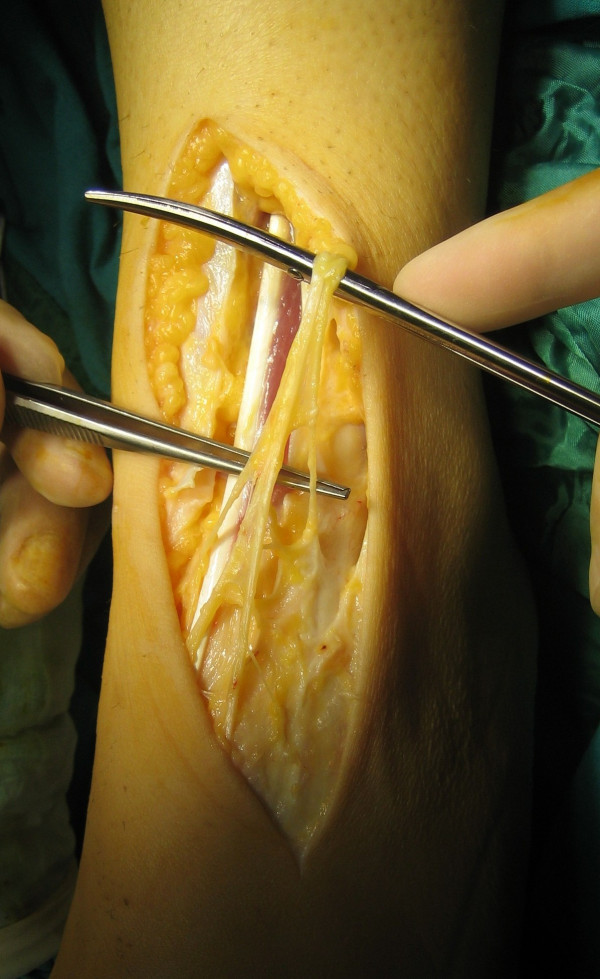
Photograph at operation showing the superficial peroneal nerve.

After preoperative assessment, the patient was admitted for surgical treatment with the diagnosis of SPN entrapment. The operation was done under general anaesthesia, using pneumatic tourniquet. Bilateral explorations of the site of tenderness revealed adhesions of both SPNs to the fascia with perineural fibrosis. Careful dissections were done to free the nerves and neurolysis was successfully performed (Figure 1). The nerves were freed distally and proximally by splitting the overlying fascia for a few centimetres above and below the site of entrapment.

Symptoms of bilateral peroneal nerve entrapment were relieved immediately and completely in the postoperative period. Physiotherapy was started immediately to prevent postoperative adhesions. No recurrence was observed in the first year following the operation.

## Discussion

Superficial peroneal nerve syndrome is an entrapment neuropathy that usually results from mechanical compression of the nerve at or near the point where the nerve pierces the fascia to travel within the subcutaneous tissue.

A thorough and accurate knowledge of the course of the SPN and its relationships is essential to understand the pathophysiology, and a thorough and careful physical examination is important for diagnosing this condition. Stephens et al. described a physical sign to identify the distal subcutaneous course of the SPN below the skin, primarily by means of plantar flexion and inversion of the ankle and foot and, secondarily by a passive flexion of the fourth toe [1].

In his study Styf, described 3 provocative tests for nerve compression at rest at rest following exercise [2]. In the first test, pressure is applied over the anterior intermuscular septum while the patient actively dorsiflexes the ankle. In the second test, the foot is passively plantar flexed and inverted at the ankle. In the third test, while the patient maintains the passive stretch, gentle percussion is applied over the course of the nerve. These tests are useful in competitive athletes who have symptoms suggestive of exercise-induced compartment syndrome.

Electrophysiological studies are helpful for the diagnosis, however, normal conduction velocity may be found especially at rest which does not exclude compression of the superficial peroneal nerve [2].

Injection of the nerve with lidocaine or Marcaine just above the site of involvement may be the most valuable diagnostic tool. The patient can define the extent of relief obtained from such an injection, which can be helpful in defining the zone of injury and expected relief from surgical release or excision.

Entrapment of the superficial peroneal nerve has traumatic and non traumatic causes. Local trauma and compression are the most common causes of nerve entrapment. This may be due to recurrent stretch injuries or certain positions like prolonged kneeling and squatting, which cause perineural fibrosis [17,18]. Oedema after trauma may result in a mini compartment syndrome which may occur when the tunnel was fibrotic, of low compliance and longer than 3 cm [2]. Chronic or exertional lateral compartment syndrome can also cause compression of the superficial peroneal nerve, particularly in athletes [19,20]. Fasciotomy of the anterior compartment for chronic anterior compartment syndrome may also cause compression of the SPN nerve [19].

Nontraumatic causes of SPN entrapment are commonly due to anatomical variations such as fascial defects, with or without muscle herniation about the lateral lower leg, where the nerve is entrapped as it emerges into the subcutaneous tissue or a short peroneal tunnel proximally. Nerve compression in patients with fascial defects is explained by the normal increase in muscle relaxation pressure and intramuscular pressure at rest during and after exercise. This increase is sufficient to cause herniated muscle tissue and this can impinge upon or compresses the nerve [20].

Lowdon reported a case of an abnormally long course of the SPN nerve through the deep fascia which was thought to have caused compression. Exercise may have exacerbated the symptoms by producing mechanical irritation or by raising the pressure in the peroneal compartment and thus increasing compression of the nerve [3].

## Conclusion

In our case, the bilateral involvement forced us to think about a systemic cause of SPN entrapment. The patient had severe loss of weight in a period of few months due to previously undiagnosed anorexia nervosa which may have caused changes in the subcutaneous tissues that led to adhesions and perineural fibrosis. Although the exact cause is unknown; SPN entrapment should be kept in mind especially in patients with severe weight loss and changes in body habits.

## Competing interests

The authors declare that they have no competing interests.
